# Accuracy of cystatin C in prediction of acute kidney injury in children; serum or urine levels: which one works better? A systematic review and meta-analysis

**DOI:** 10.1186/s12882-017-0539-0

**Published:** 2017-04-03

**Authors:** Babak Nakhjavan-Shahraki, Mahmoud Yousefifard, Neamatollah Ataei, Masoud Baikpour, Fatemeh Ataei, Behnaz Bazargani, Arash Abbasi, Parisa Ghelichkhani, Faezeh Javidilarijani, Mostafa Hosseini

**Affiliations:** 1 Pediatric Chronic Kidney Disease Research Center, Children’s Hospital Medical Center, Tehran University of Medical Sciences, Tehran, Iran; 2 Physiology Research Center and Department of Physiology, Faculty of Medicine, Iran University of Medical Sciences, Tehran, Iran; 3 Department of Pediatric Nephrology, Children’s Hospital Medical Center, Tehran University of Medical Sciences, Tehran, Iran; 4 Department of Neurology, School of Medicine, Tehran University of Medical Sciences, Tehran, Iran; 5 Department of Nuclear Medicine, Taleghani Hospital, Shahid Beheshti University of Medical Sciences, Tehran, Iran; 6 Department of Intensive Care Nursing, School of Nursing and Midwifery, Tehran University of Medical Sciences, Tehran, Iran; 7 Department of Pediatric Nephrology, Atieh Hospital, Tehran, Iran; 8 Department of Epidemiology and Biostatistics, School of Public Health, Tehran University of Medical Sciences, Poursina Ave, Tehran, Iran

**Keywords:** Cystatin C, Acute kidney injury, Prognostic value

## Abstract

**Background:**

There is still an ongoing discussion on the prognostic value of cystatin C in assessment of kidney function. Accordingly, the present study aimed to conduct a meta-analysis to provide evidence for the prognostic value of this biomarker for acute kidney injury (AKI) in children.

**Methods:**

An extensive search was performed in electronic databases of Medline, Embase, ISI Web of Science, Cochrane library and Scopus until the end of 2015. Standardized mean difference (SMD) with a 95% of confidence interval (95% CI) and the prognostic performance characteristics of cystatin C in prediction of AKI were assessed. Analyses were stratified based on the sample in which the level of cystatin C was measured (serum vs. urine).

**Results:**

A total of 24 articles were included in the meta-analysis [1948 children (1302 non-AKI children and 645 AKI cases)]. Serum (SMD = 0.96; 95% CI: 0.68-1.24; *p* < 0.0001) and urine (SMD = 0.54; 95% CI:0.34-0.75; *p* < 0.0001) levels of cystatin C were significantly higher in children with AKI. Overall area under the curve of serum cystatin C and urine cystatin C in prediction of AKI were 0.83 (95% CI: 0.80-0.86) and 0.85 (95% CI: 0.81-0.88), respectively. The best sensitivity (value = 0.85; 95% CI: 0.78-0.90) and specificity (value = 0.61; 95% CI: 0.48-0.73), were observed for the serum concentration of this protein and in the cut-off points between 0.4-1.0 mg/L.

**Conclusion:**

The findings of the present study showed that cystatin C has an acceptable prognostic value for prediction of AKI in children. Since the serum level of cystatin C rises within the first 24 h of admission in patients with AKI, this biomarker can be a suitable alternative for traditional diagnostic measures.

## Background

Acute kidney injury (AKI) is considered as one of the main public health issues all over the world. The precise prevalence of AKI in children and adolescents is unknown. However, evidences showed that the prevalence of pediatric AKI is increasing [1–4]. This disease is a risk factor for cardiovascular diseases, myocardial infarction and heart failure. The main causes of mortality in children with kidney failure are cardiovascular diseases and infections [5, 6]. AKI in children pose a great burden on the health care system and their families [7, 8]. However, recent studies have shown that preventive strategies can significantly decrease this burden [9]. Early detection and treatment can also considerably lower the costs of this health issue. Unfortunately, AKI are asymptomatic and their signs and symptoms become evident only when a great proportion of kidneys have lost their function [10]. Accordingly, all physicians in all specialties should be aware of the importance of early diagnosis and treatment of AKI in children.

Multiple diagnostic methods have been proposed for detection of AKI in children but none can accurately predict the outcome [11, 12]. In the past decade various studies have proposed measuring the serum level of certain biomarkers as a new method for early diagnosis of AKI [13–15], among which major attention has been drawn to cystatin C [16] which has been shown to be useful in diagnosing AKI and predicting its outcomes [17].

Cystatin C is a 13 kilo Dalton proteinase inhibitor from the cystatin super family of cysteine protease inhibitors which plays and important role in intra-cellular catabolism of proteins and peptides. Some studies have shown that this biomarker is a better indicator of kidney function compared to creatinine [16, 17], but still no widespread recommendations exist for its use [18]. One way to reach a general conclusion is conducting a systematic review and meta-analysis [19–22]. In this regard, Dharnidharka et al showed that cystatin C has a far greater value in assessment of glomerular filtration status compared to creatinine [16]. On the contrary, Roos et al. carried out another systematic review in 2007, the results of which showed that the diagnostic values of these two biomarkers are similar to each other [17]. In another systematic review conducted by Zheng et al. in 2011, the serum level of cystatin C was shown to be a suitable hematologic biomarker in diagnosing AKI while its urine concentration has a moderate diagnostic value for this purpose [23]. Feng et al. reviewed 6 articles and reported that an increase in cystatin C level is associated with increased risk of mortality and the need for dialysis in a 5-year follow up in patients with AKI [24]. These four meta-analyses included articles with adult sample populations, while recently a significant growth has been observed in the number of studies evaluating this matter in children, which highlight the need for conducting a meta-analysis on data gathered from children. Accordingly, the authors of the present study aimed to conduct a systematic review and meta-analysis to provide evidence on the prognostic value of cystatin C in AKI in children.

## Methods

### Search strategy

This study was designed based on the Meta-analysis of Observational Studies in Epidemiology statement [25]. The search strategy protocol and summarizing the results is presented in our previous studies in details [19–22, 26–36]. In summary, two independent reviewers carried out an extensive search in in electronic databases of Medline, ISI Web of Science, Embase, Cochrane library and Scopus until the end of 2015. Keywords were established using Mesh from the PubMed database, Emtree from the Embase database and manual search in the titles of similar articles. Search was based on the words related to cystatin C and acute kidney injury. Queries used in the databases of Medline and Embase are presented in the Table 1.

**Table 1:** Queries used for the search in Medline and Embase databases

Database	Query
Medline	("Cystatin C"[Mesh] OR "Cystatin C"[TIAB] OR "Post-gamma-Globulin"[TIAB] OR "Post gamma Globulin"[TIAB] OR "Neuroendocrine Basic Polypeptide"[TIAB] OR "Basic Polypeptide, Neuroendocrine"[TIAB] OR "Cystatin 3"[TIAB] OR "gamma-Trace"[TIAB] OR "gamma Trace"[TIAB]) AND ("Acute Kidney Injury"[Mesh] OR "Acute Kidney Injuries"[TIAB] OR "Kidney Injuries, Acute"[TIAB] OR "Kidney Injury, Acute"[TIAB] OR "Acute Renal Injury"[TIAB] OR "Acute Renal Injuries"[TIAB] OR "Renal Injuries, Acute"[TIAB] OR "Renal Injury, Acute"[TIAB] OR "Renal Insufficiency, Acute"[TIAB] OR "Acute Renal Insufficiencies"[TIAB] OR "Renal Insufficiencies, Acute"[TIAB] OR "Acute Renal Insufficiency"[TIAB] OR "Kidney Insufficiency, Acute"[TIAB] OR "Acute Kidney Insufficiencies"[TIAB] OR "Kidney Insufficiencies, Acute"[TIAB] OR "Acute Kidney Insufficiency"[TIAB] OR "Kidney Failure, Acute"[TIAB] OR "Acute Kidney Failures"[TIAB] OR "Kidney Failures, Acute"[TIAB] OR "Acute Renal Failure"[TIAB] OR "Acute Renal Failures"[TIAB] OR "Renal Failures, Acute"[TIAB] OR "Renal Failure, Acute"[TIAB] OR "Acute Kidney Failure"[TIAB] OR "Acute Kidney Tubule Necrosis"[TIAB])
Embase	'cystatin c'/exp OR 'post-gamma-globulin'/exp OR 'post gamma globulin'/exp OR 'neuroendocrine basic polypeptide'/exp OR 'basic polypeptide, neuroendocrine' OR 'cystatin 3'/exp OR 'gamma-trace'/exp OR 'gamma trace'/exp AND ('acute kidney injuries' OR 'kidney injuries, acute' OR 'kidney injury, acute' OR 'acute renal injury'/exp OR 'acute renal injuries' OR 'renal injuries, acute' OR 'renal injury, acute' OR 'renal insufficiency, acute'/exp OR 'acute renal insufficiencies' OR 'renal insufficiencies, acute' OR 'acute renal insufficiency'/exp OR 'kidney insufficiency, acute'/exp OR 'acute kidney insufficiencies' OR 'kidney insufficiencies, acute' OR 'acute kidney insufficiency'/exp OR 'kidney failure, acute'/exp OR 'acute kidney failures' OR 'kidney failures, acute' OR 'acute renal failure'/exp OR 'acute renal failures' OR 'renal failures, acute' OR 'renal failure, acute' OR 'acute kidney failure'/exp OR 'acute kidney tubule necrosis'/exp)

In order to find further articles or non-indexed data, hand-search in the reference lists of relevant studies was done. Google search engine, Google scholar and ProQuest were also manually searched.

### Selection criteria

Both retrospective and prospective, cohort and cross-sectional studies evaluating the prognostic value of cystatin C in detection of AKI in children (age < 18 years old) were included in the present study. Measuring the level of cystatin C after the final diagnosis of AKI, evaluating animals and using a reference test incompatible with standard criteria were considered as the exclusion criteria.

### Data extraction and quality assessment

The results of the search in databases were pooled and repetitive articles were removed using EndNote (version X7, Thomson Reuters Company) software. Studies were evaluated and controlled regarding their research methodology and the summary of extracted resources were assessed independently by two separate reviewers. The reason for dismissal of any articles was recorded. In case of disagreement between the two reviewers, a third reviewer studied the article and solved the disagreement through discussion (inter-rate reliability = 0.91). Results of the search were recorded in a checklist according to the guidelines designed by PRISMA statement [37]. Extracted data included information about the study setting, patients’ characteristics, definition of AKI, evaluation time, sample type (urine, serum), number of evaluated samples, outcome of the patients (progression to AKI or non-AKI) and possible bias. In cases of repetitive results, the study including the larger sample population was included. If data were not extractable from the article, the corresponding author was contacted and asked to provide the information. If data were presented as charts, the extraction method proposed by Sistrom and Mergo was utilized [38]. In cases where only sensitivity and specificity were reported, reliable online software was used for calculation of true positive (TP), true negative (TN), false positive (FP) and false negative (FN).

Quality status of the articles was assessed using the 14-item Quality Assessment of Diagnostic Accuracy Studies (QUADAS2) tool [39]. Only studies with a quality rate of good or fair were included in the sensitivity analysis.

Articles in which an appropriate reference test was not used for defining acute kidney injury were considered as having a poor quality and were excluded from the study. Good articles were the ones in which all the seven QUADAS-2 items were found to be acceptable (have low risk of bias). Even if an article had used an appropriate reference test but at least one of the other QUADAS-2 items has high risk of bias or was unclear, it was considered as having a fair quality.

### Statistical analysis

All the results of the studies were summarized into means and standard deviations, TP, FP, TN and FN. Heterogeneity between the studies was evaluated using Chi-squared and I^2^ tests and a p value of less than 0.1 was considered as statistically significant (indicative of heterogeneity) [40]. If the studies were homogenous, fixed effect model was used. In case of a positive heterogeneity, subgroup analysis was performed to identify the sources of heterogeneity. If the source of heterogeneity was not identified, random effect model was used. Eventually, all the results were pooled and an overall effect size was presented.

Analyses were carried out in two steps. In the first step the mean level of cystatin C was compared between the two groups AKI and non-AKI by calculating standardized mean difference (SMD) with a confidence interval of 95% (95% CI) based on Hedge g. in this step, publication bias was also assessed through drawing funnel plots using Egger’s and Begg’s tests [41].

In the second step, TP, FP, TN and FN cases in AKI and non-AKI patients were recorded separately for each study. These values were used for calculation of area under the curve (AUC), sensitivity, specificity, positive likelihood ratio and negative likelihood ratio with a 95% confidence interval (95% CI). Mixed-effects binary regression model, a type of random effect model was used in this section. This strategy was selected due to the significant heterogeneity between the articles. Subgroup analysis was performed to identify the sources of heterogeneity using a bivariate mixed-effects binary regression model. Deeks’ funnel plot asymmetry test was used for evaluation of publication bias.

Analyses were stratified based on the samples in which cystatin C level was measured (serum and urine). STATA version 11.0 (Stata Corporation, College Station, TX) was used for data analysis.

## Results

### Characteristics of included studies

**Fig. 1:**
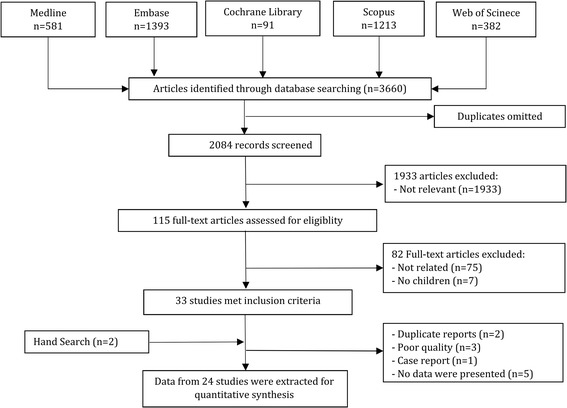
Flowchart of present systematic review and meta-analysis

After the initial screening of the search results, full texts of 115 articles were studied and a total of 24 articles were included in the meta-analysis (Fig. 1) [9, 42–64]. These articles included data from 1948 children (1302 non-AKI children and 645 AKI cases). The mean age of these children was 3.1 years old and 54.1% were boys. The most common settings were children hospitalized in the intensive care unit (ICU) in 10 studies [44, 45, 49, 52, 55, 56, 58, 59, 61, 63] and post cardiac surgery in 8 surveys [42, 50, 51, 53, 54, 57, 60, 64]. Eighteen articles measure the serum level of cystatin C [42, 43, 45, 46, 48–52, 54, 57–61, 63, 64] and five assessed the urine concentration of this protein [9, 44, 53, 55, 56]. Two studies measured the level of cystatin C in both urine and serum samples [47, 62].

AKI was defined as at least a 20% decrease in eCCl from baseline in seven studies, as 50% increase or rise in SCr of at least 0.3 mg/dL in nine studies, as the doubling of SCr from baseline in two surveys and as a GFR < 80 mL/min/1.73 m^2^ in four studies. The other four surveys had used other standard definitions for AKI. It should be mentioned that two studies had used more than one definition for classifying children with AKI. Table 2 presents the characteristics of these studies.

**Table 2:** Characteristics of included studies

Author	Country	Setting	Mean age (year)	Boys (%)	Sample size	AKI definition	Location	Storage degree^a^	Timing of CysC (hours)	Timing of SCr (hours)
Ali et al. 2013 [42]	USA	Cardiac surgery	4	47.4	19	Urine output of <0.5	Serum	NR	12	96
Al-Tonbary et al. 2004 [43]	Egypt	Cancer	5.3	63.8	47	Decrease in eCCl by at least 20% from baseline	Serum	-20	0	720
Askenazi et al. 2011 [9]	England	Low Birth Weight Infants	1	46.7	30	50% increase or rise SCr of at least 0.3 mg/dL	Urine	-20	0	48
Askenazi et al. 2012 [44]	England	ICU admitted	1	51.5	33	50% increase or rise SCr of at least 0.3 mg/dL	Urine	-20	0	48
Ataei et al. 2014 [45]	Iran	ICU admitted	1	56.1	107	Decrease in eCCl by at least 25%	Serum	-80	0	48
Benzer et al. 2015 [46]	Turkey	Contrast induced nephropathy	8.8	39.7	141	Decrease in eCCl by at least 25%	Serum	-20	0	24
Di Nardo et al. 2013 [47]	Italy	Sepsis	2.5	36.4	11	Decrease in eCCl by at least 25%	Serum and urine	-80	0 to 24	48
Elmas et al. 2013 [48]	Turkey	Respiratory distress syndrome	1	45.5	28	Cr > 1.5 mg/dL	Serum	-70	0	72
Hamed et al. 2013 [49]	Egypt	ICU admitted	7	53.1	32	GFR < 80 mL/min/1.73 m2	Serum	-20	0	24
Hassinger et al. 2012 [50]	USA	Cardiac surgery	7.6	55	100	GFR < 80 mL/min/1.73 m2	Serum	-70	0 to 24	96
Herbert et al. 2015 [51]	USA	Cardiac surgery	1	58.8	17	NGAL > 150 ng/mL	Serum	-20	24	72
Herrero-Morín et al. 2007 [52]	Spain	ICU admitted	2.9	60	25	GFR < 80 mL/min/1.73 m2	Serum	NR	24	24
Koyner et al. 2013 [53]	USA	Cardiac surgery	3.8	55	299	100% rise of Cr	Urine	-80	0 to 12	24
Krawczeski et al. 2010 [54]	USA	Cardiac surgery	3.5	53.5	374	50% increase or rise SCr of at least 0.3 mg/dL	Serum	NR	0 to 24	48
Lagos-Arevalo et al. 2014 [55]	Canada	ICU admitted	4.7	60	160	50% increase or rise SCr of at least 0.3 mg/dL	Urine	-80	0	48
Li et al. 2012 [56]	China	ICU admitted	0	54.8	62	Cr > 1.5 mg/dL	Urine	-80	Within 240	240
Mamikonian et al. 2014 [57]	USA	Cardiac surgery	1.3	43	40	Doubling of SCr from baseline	Serum	-80	2 to 24	24 to 72
Maruniak-Chudek et al. 2012 [58]	Poland	ICU admitted	0	62.5	32	50% increase or rise SCr of at least 0.3 mg/dL	Serum	-70	0 to 24	24 to 48
McCaffrey et al. 2015 [59]	UK	ICU admitted	3	53	49	Decrease in eCCl by at least 25%	Serum	-80	0	12 to 24
Peco-Antić et al. 2013 [60]	Serbia	Cardiac surgery	1.6	58	112	Decrease in eCCl by at least 25%	Serum	-80	0 to 24	48
Polat et al. 2013 [61]	Turkey	ICU admitted	8.75	44	52	50% increase or rise SCr of at least 0.3 mg/dL	Serum	-80	0	48
Sarafidis et al. 2012 [62]	Greece	Asphyxia	0	76.9	13	50% increase or rise SCr of at least 0.3 mg/dL	Serum and urine	-80	24	24 to 72
Volpon et al. 2013 [63]	Brazil	ICU admitted	3.8	53.3	122	GFR < 75 mL/min/1.73 m2	Serum	-80	0 to 24	72
Zhang et al. 2013 [64]	China	Cardiac surgery	1	69.8	43	50% increase or rise SCr of at least 0.3 mg/dL	Serum	-80	0 to 24	72

^a^Celsius, *AKI* acute kidney injury, *Cr* creatinine, *GFR* glomerular filtration rate, *eCCl* estimated creatinine clearance, *ICU* intensive care unit, *NGAL* neutrophil gelatinase-associated lipocalin, *SCr* serum creatinine

### Heterogeneity and publication bias

Analyses are presented in two separate sections evaluating the correlation between the serum level of cystatin C with occurrence of AKI and that of the urine concentration of this protein. In assessing the relation between serum level of cystatin C with AKI, a significant heterogeneity was observed between the studies (I^2^ = 91.2%; *p* < 0.001) while minor heterogeneity was found in the second part of our analyses evaluating the urine concentration of cystatin C (I^2^ = 49.1%; *p* = 0.04). In overall analysis no publication bias was found in assessment of the relation between serum level (coefficient = 0.9; 95% CI:-1.4-3.2; *p* = 0.44) and urine level (coefficient = 1.1; 95% CI:-3.9-3.3; *p* = 0.09) of cystatin C with AKI.

### Meta-analysis

#### Relation between the level of cystatin C with occurrence of AKI

In 22 studies the mean or median of cystatin C level was compared between the two groups of AKI and non-AKI children [9, 42, 44–55, 57–64].

**Fig. 2:**
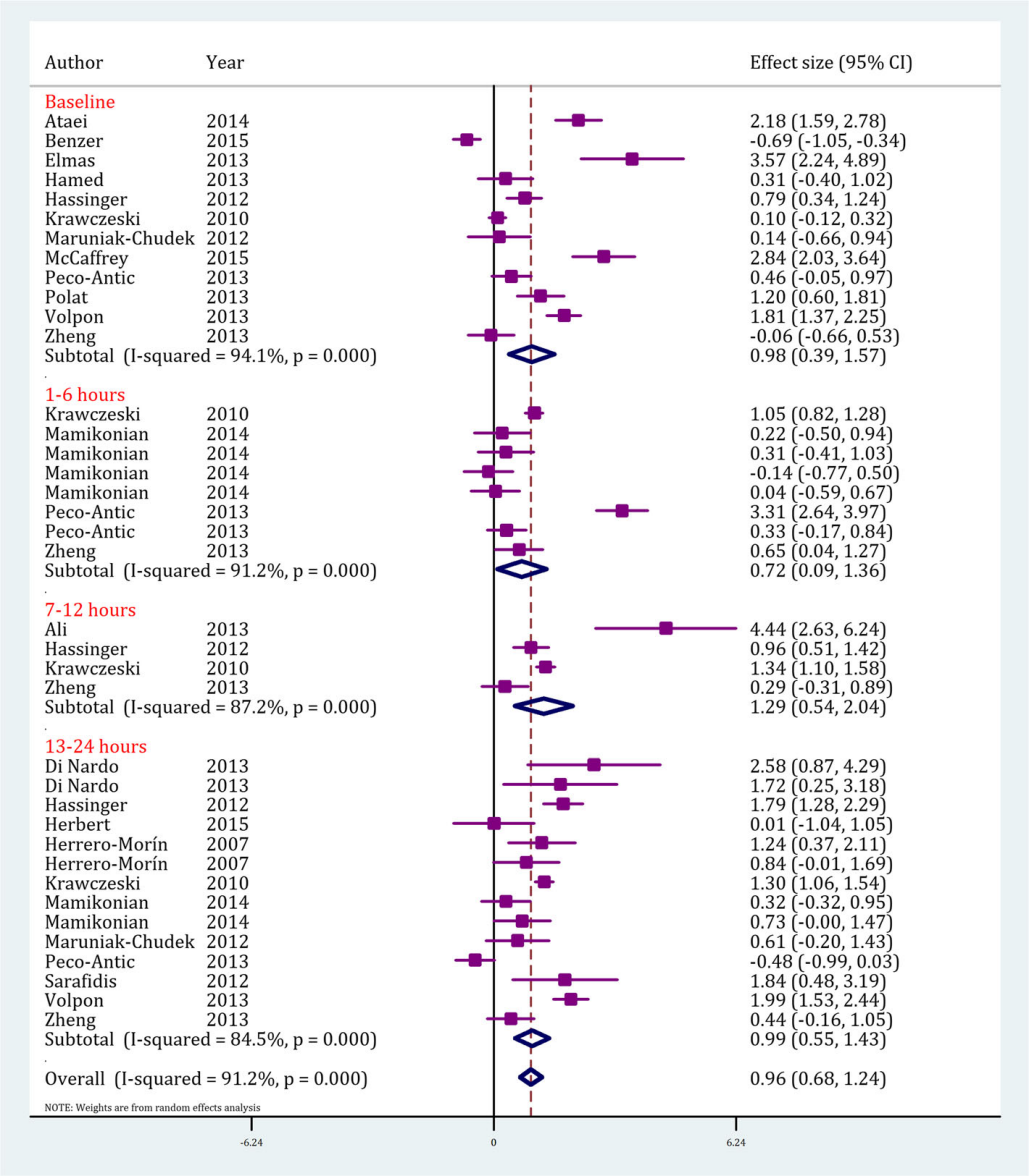
Value of serum cystatin C in prediction of acute kidney injury by time assessment. CI, confidence interval. Effect size were assessed by calculating standardized mean difference

Analyses showed that the serum level of cystatin C was significantly higher in AKI patients compared to non-AKI subjects (SMD = 0.96; 95% CI: 0.68-1.24; *p* < 0.0001). Serum cystatin C level measured on arrival (SMD = 0.98; 95% CI:0.39-1.57 l *p* < 0.0001), after 6 h (SMD = 0.72; 95% CI:0.09-1.36; *p* < 0.0001), after 12 h (SMD = 1.29; 95% CI:0.54-2.04; *p* < 0.0001) and after 24 h (SMD = 0.99; 95% CI:0.55-1.43; *p* < 0.0001) were all significantly higher in AKI patients (Fig. 2 and Table 3).

**Table 3:** Primary subgroup analyses of serum and urine level of cystatin C in patients with acute kidney injury

Characteristics	*P*_for publication bias_	Model	*P*_for heterogeneity_ (I^2^)	SMD (95% CI)	*P*_for effect size_
a) Serum level					
Timing of cystain C assessment					
Overall	0.44	REM	<0.001 (89.9%)	0.96 (0.68-1.24)	<0.001
Baseline	0.37	REM	<0.001 (91.1%)	0.98 (0.39-1.57)	<0.001
1-6 h	0.90	REM	<0.001 (91.2%)	0.72 (0.09-1.36)	<0.001
7-12 h	>0.99	REM	<0.001 (01.1%)	1.29 (0.54-2.04)	<0.001
13-24 h	0.66	REM	<0.001 (83.4%)	0.99 (0.55-1.43)	<0.001
		*Overall Significant difference among subgroups*			0.62
Timing of AKI definition					
Within 24 h	0.07	REM	<0.001 (94.6%)	0.88 (-0.40-2.17)	0.18
Within 48 h	0.60	REM	<0.001 (93.2%)	1.05 (0.62-1.15)	<0.001
Within 72 h	0.57	REM	<0.001 (76.0%)	0.74 (0.28-1.10)	0.002
More than 72 h	0.17	REM	<0.001 (86.2%)	1.58 (0.77-2.38)	<0.001
		*Overall Significant difference among subgroups*			0.44
AKI definition					
Decrease in eCCl by at least 20% from baseline	0.06	REM	<0.001 (94.5%)	1.07 (0.29-1.84)	0.007
50% increase or rise SCr of at least 0.3 mg/dL	0.58	REM	<0.001 (89.0%)	0.72 (0.37-1.08)	<0.001
GFR < 80 mL/min/1.73 m2	0.36	REM	<0.001 (78.8%)	1.25 (0.83-1.67)	<0.001
Doubling of SCr from baseline	0.34	FEM	0.61 (0.0%)	0.07 (-0.29-0.44)	0.69
Other	0.30	REM	<0.001 (92.4%)	2.61 (0.24-5.45)	0.07
		*Overall Significant difference among subgroups*			0.79
Setting					
PICU	0.69	REM	<0.001 (82.1%)	1.50 (1.02-1.99)	<0.001
Cardiac surgery	0.60	REM	<0.001 (90.5%)	0.71 (0.41-1.02)	<0.001
Other	0.03	REM	<0.001 (91.0%)	1.09 (-1.15-3.33)	0.34
		*Overall Significant difference among subgroups*			**0.02**
Sample size					
≤30	0.04	REM	<0.001 (78.0%)	1.90 (0.98-2.82)	<0.001
>30	0.91	REM	<0.001 (92.3%)	0.80 (0.50-1.10)	<0.001
		*Overall Significant difference among subgroups*			0.23
Storage degree					
-20	0.01	REM	<0.001 (90.0%)	0.82 (-0.19-1.83)	0.11
-70	0.54	REM	<0.001 (91.7%)	1.06 (0.66-1.46)	<0.001
-80	0.60	REM	<0.001 (90.3%)	0.97 (0.57-1.41)	<0.001
		*Overall Significant difference among subgroups*			**0.005**
b) Urine level					
Timing of cystain C assessment					
Overall	0.09	REM	0.04 (49.1%)	0.54 (0.34-0.75)	<0.001
Baseline	0.07	REM	0.01 (63.3%)	0.70 (0.37-1.03)	<0.001
1-12 h	0.03	FEM	0.93 (0.0%)	0.38 (0.19-0.58)	<0.001
13-24 h	0.11	REM	>0.99 (0.0%)	0.55 (-0.61-1.67)	0.36
		*Overall Significant difference among subgroups*			0.83
AKI definition					
Decrease in eCCl by at least 20% from baseline	0.32	FEM	0.82 (0.0%)	1.84 (0.78-2.89)	0.007
50% increase or rise SCr of at least 0.3 mg/dL	0.27	FEM	0.06 (52.2%)	0.56 (0.31-0.82)	<0.001
Doubling of SCr from baseline	0.32	REM	0.04 (49.1%)	0.38 (0.16-0.59)	0.69
		*Overall Significant difference among subgroups*			0.11
Timing of AKI definition					
Within 24 h	0.74	FEM	0.99 (0.0%)	0.37 (0.23-0.51)	0.18
Within 48 h	0.18	FEM	0.14 (41.7%)	1.04 (0.55-1.52)	<0.001
		*Overall Significant difference among subgroups*			0.11
Setting					
PICU	0.74	FEM	0.23 (31.1%)	0.91 (0.44-0.74)	<0.001
Cardiac surgery	0.38	FEM	0.99 (0.0%)	0.37 (0.23-0.51)	<0.001
Other	0.04	FEM	0.12 (52.2%)	1.20 (0.18-2.23)	0.02
		*Overall Significant difference among subgroups*			0.21
Sample size					
≤30	0.10	FEM	0.20 (34.7%)	0.97 (0.24-1.69)	0.009
>30	0.04	REM	0.05 (55.2%)	0.49 (0.30-0.69)	<0.001
		*Overall Significant difference among subgroups*			0.34
Storage degree					
-20	0.32	REM	0.08 (68.4%)	0.98 (-0.06-2.02)	0.11
-80	0.05	FEM	0.11 (40.7%)	0.48 (0.30-0.66)	<0.001
		*Overall Significant difference among subgroups*			0.26

*AKI* acute kidney injury, *CI* confidence interval, *FEM* fixed effect model, *REM* random effect model, *SMD* standardized mean difference, Bold values are significant. Significance among subgroups were calculated based on univariate meta-regression analysis

It seems that the setting of the study affects the serum level of cystatin C in patients. As presented in Table 3 the serum level of cystatin C in children with AKI admitted to the pediatric intensive care unit (SMD = 1.50; 95% CI: 1.02-1.99; *p* < 0.001) and the ones who developed AKI following cardiac surgery (SMD = 0.71; 95% CI: 0.41-1.02; *p* < 0.001) was significantly higher than non-AKI children while in other settings (including sepsis, cancer, Low Birth Weight Infants, Contrast induced nephropathy, Contrast induced nephropathy and Asphyxia) no significant difference was observed between AKI and non-AKI children (SMD = 1.09; 95% CI: -1.15-3.33; *p* = 0.34). The temperature at which serum was stored before conducting protein assays was another factor affecting the prognostic value of serum cystatin C level. The results showed a significant difference in the levels of cystatin C between AKI and non-AKI children when their serum were stored at the temperatures of -70 °C (SMD = 1.06; 95% CI: 0.66-1.46; *p* < 0.001) and -80 °C (SMD = 0.97; 95% CI: 0.57-1.41; *p* < 0.001) but such association was not observed in patients whose sera were stored at -20 °C (SMD = 0.82; 95% CI: -0.19-1.83; *p* = 0.11).

Timing of AKI definition (*p* = 0.44), AKI definition criteria (*p* = 0.79), and sample size of study (*p* = 0.23) had no significant effects on the difference between AKI and non-AKI children regarding their reported serum levels of cystatin C in the serum.

**Fig. 3:**
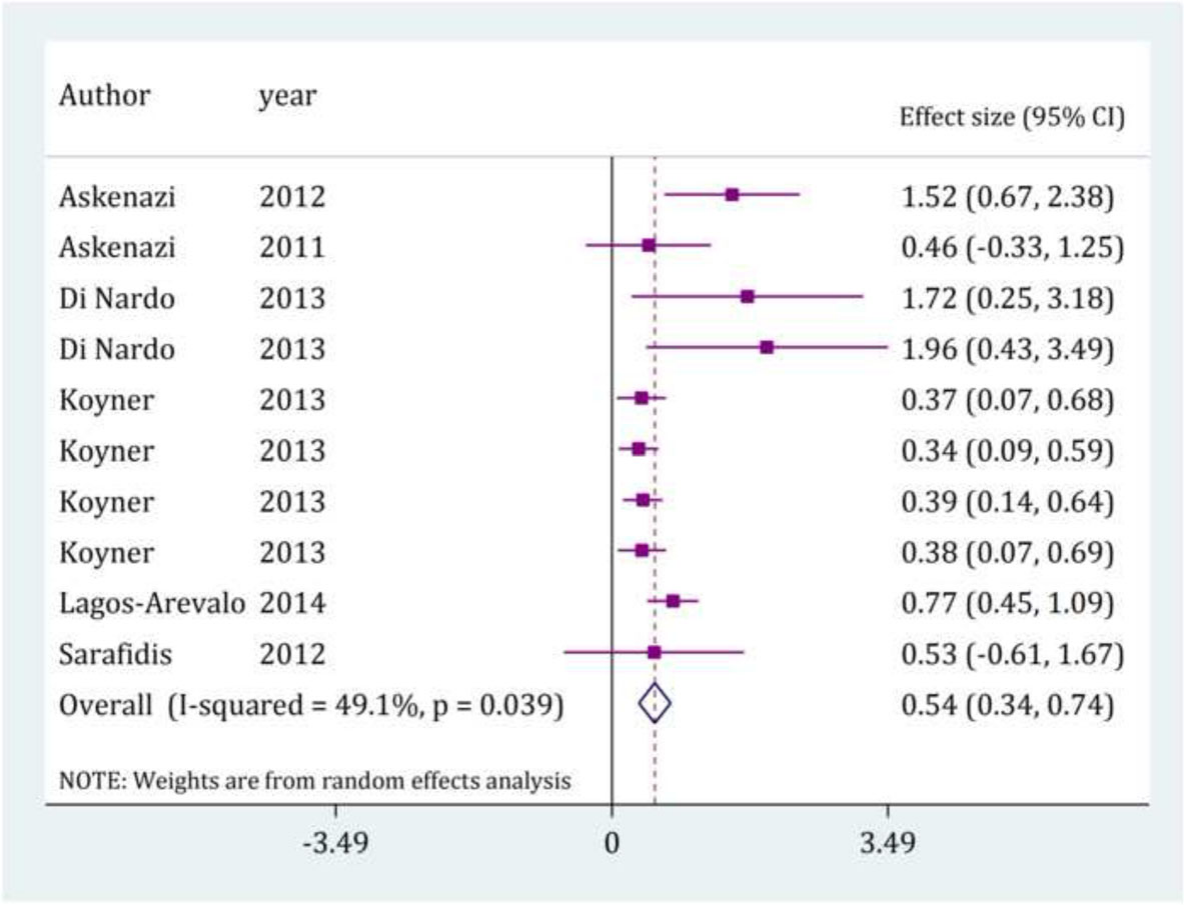
Value of urine cystatin C in prediction of acute kidney injury by time assessment. CI, confidence interval. Effect size were assessed by calculating standardized mean difference

The urine level of this protein was also found to be higher in children with AKI (SMD = 0.54; 95% CI: 0.34-0.75; *p* < 0.0001). Cystatin C levels on arrival (SMD = 0.70; 95% CI: 0.37-1.03; *p* < 0.0001) and after 12 h (SMD = 0.38; 95% CI: 0.19-0.58; *p* < 0.0001) were significantly higher in AKI patients but the differences in its levels between AKI and non AKI subjects after 24 h (SMD = 0.53; 95% CI:-0.61-1.67; *p* = 0.36) were not statistically significant (Fig. 3 and Table 3).

In addition, timing of AKI definition (*p* = 0.11), AKI definition criteria (*p* = 0.11), sample size of study (*p* = 0.34), storage degree (*p* = 0.26) and setting of study (*p* = 0.21) did not affect the difference in urine cystatin C levels between AKI and non-AKI children.

As can be seen, a significant heterogeneity was found between the studies. Subgroup analysis and meta-regression was carried out in order to find the sources of heterogeneity. This analysis revealed that the most important source of heterogeneity in assessment of serum cystatin C level were the setting of the studies and storage degree of serum. Results of this section are presented in Table 4.

**Table 4:** Multivariate meta-regression analysis for assessment of source of heterogeneity in serum level of cystatin C

Variable	Odds ratio	95% CI	P
Timing of Cystatin C			
Baseline	*Ref.*	*Ref.*	---
1-6 h	2.34	0.86-6.34	0.09
7-12 h	1.92	0.60-6.15	0.26
13-24 h	1.52	0.65-3.56	0.32
Timing of AKI definition			
Within 24 h	*Ref.*	*Ref.*	---
Within 48 h	0.44	0.07-3.00	0.39
Within 72 h	0.16	0.02-1.46	0.10
More than 72 h	0.58	0.05-6.8	0.65
AKI definition			
Other	*Ref.*	*Ref.*	---
Decrease in eCCl by at least 20% from baseline	0.08	0.002-2.32	0.13
50% increase or rise SCr of at least 0.3 mg/dL	0.06	0.004-1.07	0.09
GFR < 80 mL/min/1.73 m2	0.07	0003-1.97	0.12
Doubling of SCr from baseline	0.11	0.01-1.3	0.08
Setting			
Other	*Ref.*	*Ref.*	---
Cardiac surgery	2.52	0.45-14.17	0.24
PICU admitted	5.45	1.23-24.10	**0.03**
Sample size			
≤30	*Ref.*	*Ref.*	---
>30	0.43	0.10-1.80	0.23
Storage degree			
-20	*Ref.*	*Ref.*	---
-70	10.03	1.61-62.27	**0.02**
-80	11.69	1.13-121.08	**0.04**

*AKI* acute kidney injury, *CI* confidence interval, *PICU* pediatric intensive care unit, Bold values are significant

Since only 6 studies were included in the analyses on urine cystatin C level, meta-regression could not be performed due to statistical limitations.

### Diagnostic performance characteristics of cystatin C in AKI

**Fig. 4:**
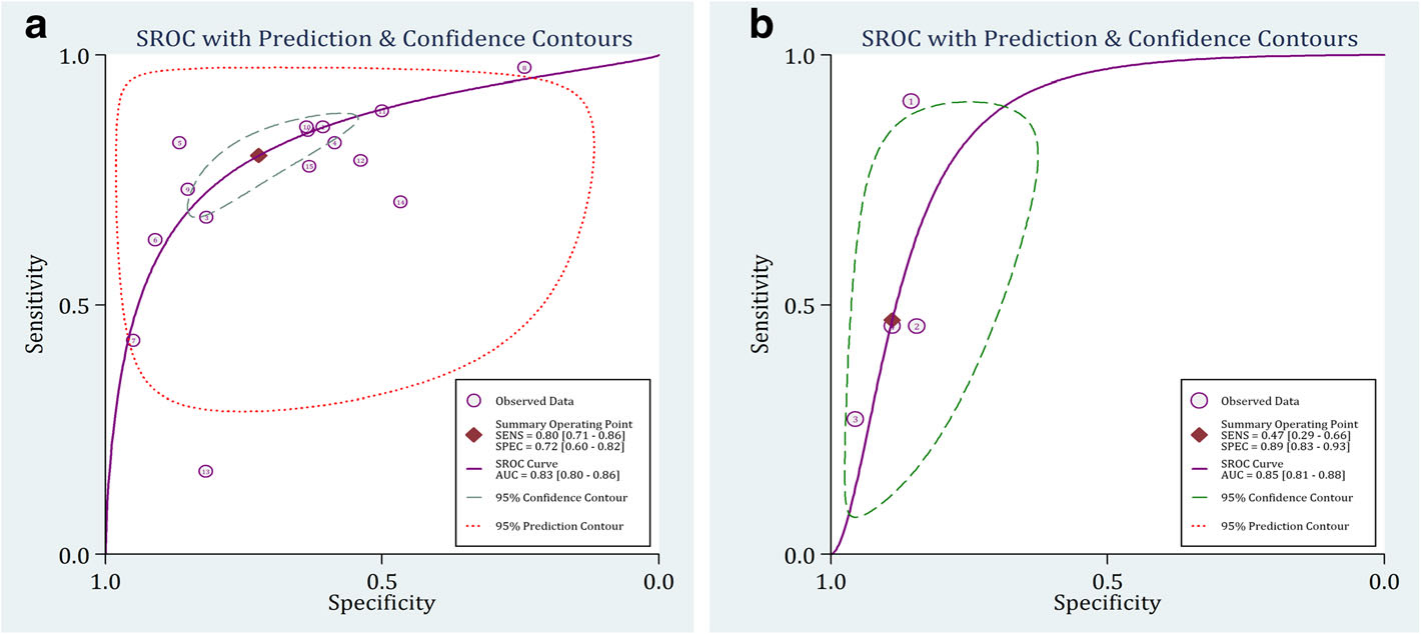
Summary receiver operative curves (SROC) for cystatin C in detection of acute kidney injury. **a** Serum; **b** Urine. AUC: Area under the curve; SENS: Sensitivity; SPEC: Specificity

Table 5 presents the sensitivity, specificity and diagnostic likelihood ratio of the level of cystatin C protein in detection of AKI in different cut off points. Overall AUC of serum cystatin C in prediction of AKI was 0.83 (95% CI: 0.80-0.86) (Fig. 4-a). The area under the curve in cut-off points of 0.4-1.0 mg/L and 1.01-2.5 mg/L were 0.83 (95% CI: 0.80-0.86) and 0.85 (95% CI: 0.82-0.88), respectively. Overall area under the curve of urine cystatin C in prediction of AKI was 0.85 (95% CI: 0.81-0.88) (Fig. 4-b). Since only four studies were included in urine level analysis, subgroup analysis was not performed. There was no significant difference between AUC of serum and urine levels of cystatin C in prediction of AKI (*p* = 0.25)

**Table 5:** Diagnostic performance characteristics of cystatin C in detection of acute kidney injury

Characteristics	TP	FP	FN	TN	P _for publication bias_	Model	P _for heterogeneity_ (I^2^)	Effect size (95% CI)
a) Serum level	543	445	206	1102				
Overall area under the curve					0.08	REM	<0.001 (80.0)	0.83 (0.80-0.86)
0.4-1 mg/L	314	361	53	386				
Sensitivity					0.29	REM	<0.001 (77.6)	0.85 (0.78-0.90)
Specificity					0.29	REM	<0.001 (94.6)	0.61 (0.48-0.73)
Positive likelihood ratio					0.29	REM	<0.001 (92.8)	2.18 (1.66-2.88)
Negative likelihood ratio					0.29	REM	0.01 (60.3)	0.24 (0.18-0.34)
1.01-2.5 mg/L	229	84	153	716				
Sensitivity					0.01	REM	<0.001 (87.1)	0.65 (0.49-0.77)
Specificity					0.01	REM	<0.001 (87.8)	0.88 (0.79-0.94)
Positive likelihood ratio					0.01	REM	<0.001 (64.5)	5.56 (3.62-8.53)
Negative likelihood ratio					0.01	REM	<0.001 (85.5)	0.40 (0.28-0.56)
b) Urine level	93	37	128	295				
Overall area under the curve					0.07	REM	<0.001 (83.3)	0.85 (0.81-0.88)
Sensitivity					0.07	REM	<0.001 (83.3)	0.47 (0.29-0.66)
Specificity					0.07	REM	0.09 (54.3)	0.89 (0.83-0.93)
Positive likelihood ratio					0.07	FEM	0.24 (0.0)	4.24 (2.93-6.13)
Negative likelihood ratio					0.07	REM	0.04 (63.2)	0.60 (0.43-0.83)

*AKI* acute kidney injury, *CI* confidence interval, *FEM* fixed effect model, *REM* random effect model, *TP* true positive, *TN* true negative, *FP* false positive, *FN* false negative

The best sensitivity and specificity were observed for the serum concentration of cystatin C and in the cut-off points between 0.4-1.0 mg/L, calculated to be 0.85 (95% CI:0.78-0.90) and 0.61 (95% CI:0.48-0.73), respectively.

## Discussion

The present study aimed to gather information on the predictive value of cystatin C for AKI in children. The results for both urine and serum concentrations of this biomarker showed that cystatin C has an acceptable prognostic value for prediction of AKI in children. We included studies that had evaluated the serum or urine level of cystatin C in the first 24 h after admission or surgery of the patients. In most of these studies AKI had been diagnosed by conventional biomarkers such as serum creatinine level in the second, third or fourth day (24-96 h) post-admission or surgery. So it seems that cystatin C rises earlier than serum creatinine in response to AKI and can be a potential substitute for creatinine concentration.

It seems that the serum level of cystatin C has a higher predictive value for AKI compared to its urine concentration. However, the number of studies with cystatin C urine concentration assessment was limited [9, 44, 47, 53, 55, 62], which caused the p value of publication bias evaluation to be borderline (p = 0.09). In agreement with these findings, two other meta-analyses had shown the superiority of serum cystatin C levels to its urine concentrations for predicting the progression of AKI [16, 23]. Following an AKI the serum level of this protein rises earlier than its urine level and in order for the cystatin C urine concentration to rise, tubular injury should occur [65], while in a considerable number of these patients, tubular injury does not occur in the first stages of AKI. In addition, a substantial portion of the cystatin C that is filtered at the glomerulus is metabolized by the kidney [66]. Hence, it is anticipated that the urine concentration of cystatin C rises later than its serum levels in response to AKI.

Two factors should be evaluated when the prognostic value of a biomarker in prediction of AKI is being assessed. The first one is the time of assessment and the second is the cut-off point. A suitable biomarker should be able to diagnose a condition in the shortest amount of time with a high accuracy [31, 67]. In available literature on this subject, the level of cystatin C was assessed at different times (from arrival to 72 h after the surgery or admission); however, since time plays an important role in prognosis of AKI patients we decided to include data in which the level of cystatin C was measured in the first 24 h after arrival of the patient (or after surgery). The results showed that the relation between the urine or serum level of cystatin C with occurrence of AKI is not affected by the time in which the concentration is measured in the first 24 h. So, it can be concluded that assessing the level of cystatin C in the first 24 h can predict occurrence of AKI in the following days and its prognostic value does not significantly differ whether its concentration is measured on arrival or after 24 h. In this regard, it is suggested that a serum sample should be drawn on arrival for measuring the level of cystatin C in order to assess the risk of AKI development.

Moreover, it should be noted that in the majority of included studies in this meta-analysis, AKI was diagnosed based on the rise in serum creatinine level between 24 to 96 h post-admission or surgery while the findings of this survey showed that serum cystatin C levels measured in the first 24 h, can predict development of AKI in the next few days. So it seems that cystatin C levels respond to AKI earlier than the conventional biomarkers such as creatinine and urine output, one of the strengths of this biomarker compared to others. To confirm this hypothesis, direct comparison between cystatin C and creatinine levels could not be performed due to the limited number of studies which had included this direct comparison in their analyses. Nevertheless, AKI was defined based on serum creatinine levels in the included studies and so an indirect comparison has already been conducted between the two biomarkers.

The cut off points also differed between included studies (ranging from 0.4 mg/L to 2.5 mg/L) so we categorized these articles into two groups of a cut-off point less than 1.0 mg/L and a cut-off point off higher than 1.0 mg/L (serum level). The cut-off level of 1 mg/L was selected based on the previous studies showing that the normal serum level of this biomarker in children aged 1 to 17 is always less than this level [68].

The extensive search in electronic databases and using hand-search to yield maximum relevant articles add to the strength of the present study. Another strength of this survey was the subgroup analysis performed based on two important factors of measuring time and cut-off point. The significant heterogeneity observed between the included studies was one of the weaknesses in this survey, the source of which was identified to be the setting of these studies. Moreover, in assessing the diagnostic value of cystatin C urine concentration, only six studies were included which makes the yielded results susceptible to publication bias. It should also be mentioned that all the included studies were observational surveys which increases the possibility of selection bias in these studies.

Finally, it should be mentioned that the definition of AKI differed between the studies which might have affected the results of the survey. On the other hand, AKI had been diagnosed at different times (between 24 to 720 h) which made interpretation of the findings quite difficult and their generalization doubtful. However, subgroup analysis and meta-regression showed that the differences in definition and timing of AKI do not affect the prognostic value of cystatin C in predicting development of AKI.

## Conclusion

The present meta-analysis is the first to assess the prognostic value of cystatin C in detection of AKI in pediatric population. The findings of this study showed that cystatin C has an acceptable prognostic value for prediction of AKI in children, with its serum concentration diagnostic value being higher than that of its urine level. So, measuring cystatin C serum level in the first 24 h and considering a cut-off point between 0.4-1.0 mg/L provides the highest value in predicting AKI.

## Abbreviations

AKI: Acute kidney injury
CI: Confidence interval
SMD: Standardized mean difference
